# Porcine Bocavirus Infection Associated with Encephalomyelitis in a Pig, Germany^1^

**DOI:** 10.3201/eid2207.152049

**Published:** 2016-07

**Authors:** Vanessa M. Pfankuche, Rogier Bodewes, Kerstin Hahn, Christina Puff, Andreas Beineke, André Habierski, Albert D.M.E. Osterhaus, Wolfgang Baumgärtner

**Affiliations:** University of Veterinary Medicine, Hannover, Germany (V.M. Pfankuche, K. Hahn, C. Puff, A. Beineke, A. Habierski, A.D.M.E. Osterhaus, W. Baumgärtner);; Center for Systems Neuroscience, Hannover (V.M. Pfankuche, K. Hahn, A. Beineke, W. Baumgärtner);; The Erasmus University Medical Center, Rotterdam, the Netherlands (R. Bodewes);; Utrecht University, Utrecht, the Netherlands (R. Bodewes)

**Keywords:** central nervous system, encephalomyelitis, next generation sequencing, *in situ*-hybridization, parvovirus, porcine bocavirus, viruses, pigs, Germany

**To the Editor:** In 2013, a 6-week-old female piglet kept in a flatdeck cage had coughing, growth retardation, and diarrhea and was taken to a local veterinarian in Hannover, Germany; the piglet was euthanized. After necropsy at the University of Veterinary Medicine in Hannover, histologic investigation found interstitial pneumonia; a mild, multifocal, lymphohistiocytic panencephalitis that affected the cerebrum and cerebellum, including brain stem and medulla oblongata; and a mild, multifocal, lymphohistiocytic panmyelitis. Results from screening for typical neurotropic viruses (classical swine fever virus, suid herpesvirus 1, rabies virus, teschovirus, porcine enterovirus 8, 9, and 10) were negative; *Mycoplasma hyorhinis* was detected by multiplex PCR (Institute of Virology, University of Veterinary Medicine Hannover) within the lung and pulmonary lymph nodes. Cerebral tissue from the pig was processed for viral metagenomics by random RNA and DNA virus screening and next-generation sequencing (NGS) with the 454 sequencing platform (GS Junior; Roche, Basel, Switzerland), as described (*1*), and 21,359 reads were obtained. Analysis by using blastn and blastx (*2*) showed 10 reads had >97% nt identity with porcine bocavirus (PBoV) KU14. No other viral sequences were detected.

By using primers based on sequence data of the PBoV, partially overlapping PCR amplicons were obtained to confirm and extend the NGS data of the isolate, which was named PBoV S1142/13 (*1*; GenBank accession no. KU311698). A total of 2,176 nt of PBoV S1142/13 were obtained, consisting of the partial nucleoprotein (NP) 1 and the nearly complete viral protein (VP) 1 gene. By using MAFFT version 7 (http://mafft.cbrc.jp/alignment/server/), we aligned the nearly complete VP1 gene of PBoV S1142/13 with various closely related members of the genus *Bocaparvovirus* and built a maximum-likelihood tree by using the general time reversible plus invariable sites plus gamma distribution method, as determined by jModelTest 2.0 (*3*) and default parameters in MEGA6.06 (*4*). Results confirmed that PBoV S1142/13 was most closely related to PBoV KU14 (Figure, panel A). The partial genome of PBoV S1142/13 differed at 8 nt positions from PBoV KU14, resulting in 99.6% nt identity. Of these nucleotide differences, 4 resulted in an amino acid difference, including position 2733 (T→C on the basis of PBoV KU14 as a reference genome), which is part of the NP1 stopcodon of PBoV KU14. These results indicate that the stopcodon was located 39 nt farther downstream than for PBoV KU14. The other 3 aa differences were present in the VP1 protein; each of these differences was within the same group of amino acids as those detected in PBoV KU14.

For further substantiation of a potential cause-effect relationship of histologic (Figure, panel B) and NGS results, we performed fluorescent in situ hybridization (FISH) on formalin-fixed, paraffin-embedded central nervous system (CNS) sections of the diseased animal and of a control pig with no CNS lesions. We used an RNA probe specific for the obtained NP1 and VP1 sequences covering 1,153 nt (Affymetrix, Santa Clara, CA, USA) according to the manufacturer’s protocol, with minor variations (ViewRNA ISH Tissue 1-Plex Assay Kit and ViewRNA Chromogenic Signal Amplification Kit, Affymetrix). A probe specific for porcine ubiquitin (*Sus scrofa* ubiquitin C; GenBank accession no. XM_005657305; nt 2–890) served as a positive control.

The spinal cord of the diseased pig showed diffuse intracytoplasmic and intranuclear PBoV-specific signals within scattered neurons adjacent to the histologically detected inflammatory lesions (Figure, panel C). The negative control and the nonprobe incubation lacked PBoV-specific signals. The porcine ubiquitin probe provided a strong intracellular and extracellular staining within the CNS of both pigs.

**Figure:**
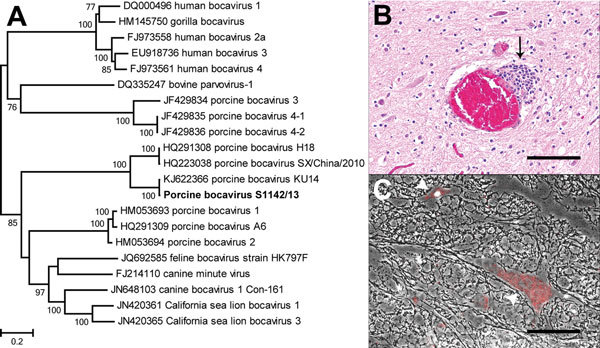
Phylogenetic analysis and staining of porcine bocavirus (PBoV) from the spinal cord of a diseased pig, Hannover, Germany. A) Phylogenetic relationship of PBoV isolate S1142/13 (bold) with other bocaviruses. The nucleotide sequence of the nearly complete viral protein 1 of PBoV S1142/13 was aligned with other members of the genus *Bocaparvovirus*, and a maximum-likelihood phylogenetic tree was prepared by using the general time reversible plus invariable sites plus gamma distribution model and 500 bootstrap replicates. Only bootstrap values >70 are shown. Scale bar indicates nucleotide substitutions per site. B) Spinal cord of the diseased pig showing perivascular accentuated mild, focal, nonsuppurative inflammation (arrow). Hematoxylin and eosin stain. Scale bar indicates 100 µm. C) Intracytoplasmic and intranuclear PBoV-specific red positive signals in neurons of the spinal cord of the diseased pig (arrowheads), determined by using fluorescent in situ hybridization (Fast Red; ViewRNA Chromogenic Signal Amplification Kit; Affymetrix, Santa Clara, CA, USA). Phase contrast and fluorescent microscopy. Scale bar indicates 100 µm.

PBoV (genus *Bocaparvovirus*, family *Parvoviridae*) was first described in 2009 as porcine boca-like virus in pigs in Sweden with postweaning multisystemic wasting syndrome (*5*). PBoV is usually involved in respiratory and intestinal diseases in pigs (*5*) but has not been detected in the CNS. In the pig in our study, the lack of detection of other viral sequences by using NGS indicates the potential role of PBoV as a pathogen that triggers encephalomyelitis. FISH substantiated the NGS results and revealed neuronal intracytoplasmic and intranuclear PBoV-specific signals adjacent to the lesion, indicating intraneuronal transcription and replication (*6*). Nevertheless, a potential synergistic effect of *M. hyorhinis* on the PBoV pathogenesis cannot be ruled out. Similarly, co-infection of *M. hyorhinis* and porcine circovirus type 2 has been associated with enhanced inflammatory lesions in the lungs of pigs (*7*).

The CNS tropism of PBoV S1142/13 could result from various factors, including specific amino acid changes that enable the virus to pass the blood–brain barrier and infect neurons. Additional studies are necessary to elucidate a possible role of the amino acid differences between PBoV S1142/13 and PBoV KU14 in the tropism of these viruses.

Human bocavirus has recently been found in the cerebrospinal fluid of patients having encephalitis (*8*), and related human parvovirus 4 (*9*) and human parvovirus B19 (*10*) have been reported in human encephalitis. The correlation of PBoV-specific signals by using FISH for histologic detection of encephalomyelitis assigns PBoV a potential role in provoking CNS lesions. PBoV should be considered as a cause of encephalomyelitis but needs further investigation.
